# The X‐Ray Crystal Structure of BorF, the Flavin Reductase Subunit of a Two‐Component Flavin‐Dependent Tryptophan Halogenase

**DOI:** 10.1002/prot.70131

**Published:** 2026-03-18

**Authors:** Zheng Ma, Emily W. Rady, Aravinda J. de Silva, John J. Bellizzi

**Affiliations:** ^1^ Department of Chemistry and Biochemistry College of Natural Sciences and Mathematics, The University of Toledo Toledo Ohio USA

**Keywords:** biosynthesis, crystallography, flavin adenine dinucleotide, oxidoreductase

## Abstract

BorF is a short‐chain flavin reductase from a desert soil bacterium that uses NADH to reduce FAD to FADH_2_, which is used by the tryptophan‐6‐halogenase BorH to chlorinate tryptophan in the biosynthetic pathway of borregomycin A. The X‐ray crystal structure of BorF bound to FAD was solved to 2.37 Å by molecular replacement. It consists of a homodimer of single‐domain protomers, each with a Greek key split β‐barrel topology containing a domain‐swapped N‐terminal α‐helix, as previously seen in homologous proteins. Insertions and deletions in the region between α3 and β5 result in different conformations of the adenosine portion of FAD bound to BorF and structurally related reductases. Comparison of the FAD‐bound structures of BorF and BorH suggests that FAD must completely dissociate from BorH in order to be reduced by BorF.

## 1 Introduction

Flavin‐dependent halogenases (FDHs) use FADH_2_, O_2_, and Cl^−^ to generate HOCl, which travels down a 10 Å tunnel to halogenate aromatic substrates via electrophilic aromatic substitution [1, 2, 3, 4] (Figure S1). These bacterial and fungal biosynthetic enzymes are attractive for green chemistry applications because they regioselectively synthesize aryl halides under mild reaction conditions. This facilitates atom‐efficient production of bioactive halogenated compounds and synthetically useful intermediates for cross‐coupling reactions [5, 6, 7]. FADH_2_ is oxidized to FAD during HOCl formation, but FDHs cannot regenerate FADH_2_ to complete the catalytic cycle. Instead, the FAD is released and this function is “outsourced” to a flavin reductase (FR) partner protein. FDH/FR pairs are a subclass of the two‐component flavin‐dependent monooxygenases, in which flavin acts as a diffusible cosubstrate/product for the two proteins, with the FDH subunit accepting FADH_2_ as a substrate and releasing FAD as a product and the FR subunit reducing FAD to FADH_2_ using NADH (Figure S1) [8, 9].

**FIGURE 1:**
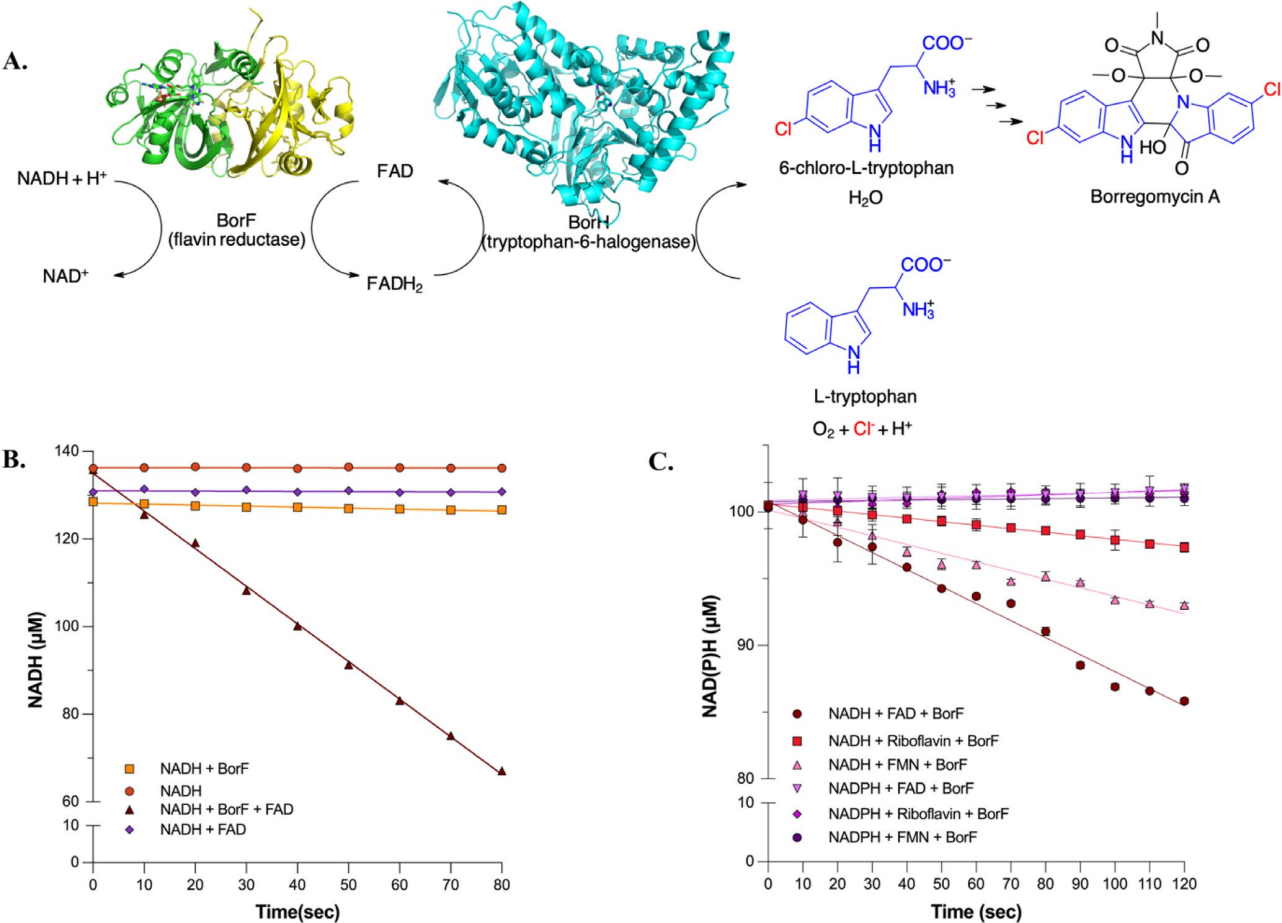
BorF is a flavin reductase. (A) BorF supplies reduced flavin to BorH, which chlorinates Trp in the biosynthetic pathway producing borregomycin A. (B) Oxidation of NADH (monitored by absorbance at 340 nm) requires both FAD and BorF. (C) BorF requires NADH (not NADPH) as reductant. Oxidation of NADH can be observed in the presence of FAD, FMN, and riboflavin, but is fastest with FAD.

Borregomycin A (Figure 1A) is a chlorinated bisindole alkaloid synthesized via a biosynthetic pathway encoded by a gene cluster from a soil bacterial metagenome from the Anza‐Borrego desert [10]. Work on related natural products has shown that the indolotryptoline core is assembled via oxidative dimerization of tryptophan, and the chlorine is incorporated before dimerization by chlorination of tryptophan [11, 12]. The borregomycin A gene cluster includes two genes, *borH* and *borF*, which encode proteins with homology to the FDH and FR subunits of a two‐component system [10]. We have previously shown that BorH chlorinates and brominates tryptophan at C6 in vitro, and can halogenate other aromatic compounds containing indole, benzene, and quinoline groups [13, 14]. BorF and NADH are required for BorH to halogenate Trp, implicating BorF as the FR that partners with BorH (Figure 1A) [15]. BorH and BorF exhibit enhanced thermal stability (*T*_*m*_ of 48°C for BorH and 50°C for BorF), reflecting their origins from desert soil bacteria [13]. FDH/FR systems catalyze industrially useful halogenation reactions [16, 17, 18]. Thermostable enzymes are especially useful for such applications because they have extended catalytic lifetimes and can tolerate higher temperatures where rates are faster [19, 20]. Naturally occurring and engineered thermostable FDHs and FRs have enhanced catalytic properties [21, 22, 23, 24, 25], but the structural determinants underlying their enhanced stability are poorly understood.

BorF belongs to a family of short‐chain oxidoreductases (InterPro IPR002563; Pfam PF01613; SMART SM00903), which includes other FRs that supply reduced flavin to two‐component monooxygenases as well as enzymes that reduce cobalamin and iron. Crystal structures of reductases in this family [26, 27, 28, 29, 30, 31, 32, 33, 34] have captured complexes with bound FAD or FMN, and some have had nicotinamide cosubstrate/coproduct bound with the flavin in a ternary complex. These short chain FRs bind the nicotinamide dinucleotide cosubstrate in a compact folded conformation in which the nicotinamide ring is sandwiched between the flavin isoalloxazine and the NADH adenine (Figure S2A) [26, 28]. The juxtaposition of the nicotinamide and isoalloxazine enables hydride transfer from C4 of NADH to N5 of FAD (Figure S1). This binding mode differs significantly from the extended conformation of NAD(P)/NADPH seen in oxidoreductases with the canonical Rossmann fold super‐secondary structure, which is the most common dinucleotide binding architecture [35].

Previous work on FRs from two‐component flavin‐dependent monooxygenase and halogenase systems has revealed diverse kinetic mechanisms and flavin transfer mechanisms despite high sequence and structural homology. For example, BorF binds FAD before NADH in an ordered sequential kinetic mechanism, whereas AbeF uses a random sequential mechanism, and PheA2 uses a ping‐pong bisubstrate mechanism involving a cofactor flavin and a substrate flavin [15, 26].

Enzymes in this class bind and release flavin from solution, allowing any FDH to work with any FR, and most two‐component systems appear to rely on free diffusion of flavin between subunits [8, 36]. However, some two‐component systems may have evolved mechanisms to efficiently transfer the flavin between subunits, such as protein–protein interactions, allosteric regulation of reduced flavin dissociation from the reductase, and the formation of a transient FAD‐bridged flavin transfer complex [32, 36, 37, 38, 39]. Such systems may have evolved to improve efficiency, since freely diffusing FADH_2_ may react with O_2_ or other oxidants in solution. As a result, even though in vitro halogenation of substrates can be accomplished by FDHs using any source of FADH_2_, it has been postulated that the efficiency of the flavin transfer may be enhanced by using the specific FR that co‐evolved with the FDH.

To further our understanding of the BorH‐BorF two‐component FDH/FR system, we have determined the crystal structure of BorF complexed with FAD. The only previously published crystal structure of an FR known to provide FADH_2_ to a halogenase is SgcE6, part of the biosynthetic pathway producing the enediyne antitumor antibiotic C‐1027. SgcE6 has been shown to supply free reduced flavin to both a monooxygenase (SgcC) and halogenase (SgcC3), both of which modify an acyl carrier protein‐linked tyrosine substrate [33, 40]. The crystal structure of SgcC3 has not been solved, making BorF/BorH the first two‐component halogenase for which both the FR and FDH subunits have had experimentally determined structures. Our results suggest that BorH and BorF rely on diffusible flavin rather than formation of a stable protein–protein complex.

## 2 Materials and Methods

### 2.1 Macromolecule Production

The BorF amino acid sequence (GenBank: AGI62216.1, Uniprot M9QXS1, Table S3) [10] was used to design a synthetic *borF* gene (ThermoFisher GeneArt). This sequence was amplified by PCR and inserted by Gibson assembly cloning (New England Biolabs) into vector p28‐His_G_, which encodes an N‐terminal His_6_‐maltose binding protein fusion tag with a PreScission protease cleavage site. His_6_‐MBP‐BorF was overexpressed in *Escherichia coli* Rosetta2 (DE3) pLysS cells (Novagen) in Luria Broth (Research Products International) with 50 μg/mL kanamycin and 30 μg/mL chloramphenicol. A 250 mL flask containing 40 mL media and antibiotics was inoculated with a single colony and grown at 37°C with 250 rpm shaking for 16 h overnight. The next day, 5 mL of this overnight starter culture was diluted into two 2 L baffled culture flasks, each containing 500 mL of fresh LB/kanamycin/chloramphenicol, for a total culture volume of 1 L. The cultures were grown at 37°C with 250 rpm shaking until an OD_600_ of 0.6 was reached, at which point the temperature was lowered to 16°C and cultures were allowed to cool down without shaking for 30 min. The cells were then induced with 100 μM IPTG. After 20 h post‐induction growth at 16°C, cells were harvested by centrifugation at 4°C and 12 000 × *g*. Cell pellets were resuspended in 20 mM Tris pH 7.5, 500 mM NaCl, 1 mM TCEP, with an EDTA‐free protease inhibitor tablet (Roche), and incubated on ice for 30 min. The cell suspension was lysed by sonication (Fisher Scientific Sonic Dismembrator 500 at 30% power, 20 s on 20 s off for 6 min total), and the sonicated lysate was clarified by centrifugation at 12 000 × *g* for 30 min at 4°C.

His_6_‐MBP‐BorF was captured from the clarified lysate by affinity chromatography using an MBPTrap HP 5 mL column on an AktaPurifier 10 system (GE Healthcare). The column was washed with 20 mM Tris, 100 mM NaCl pH 7.5 and His6‐MBP‐BorF was eluted with 20 mM Tris, 100 mM NaCl, 10 mM maltose pH 7.5. Pooled His_6_‐MBP‐BorF fractions were treated with 100 U of PreScission protease at 4°C for 16 h to cleave the His_6_‐MBP from BorF. The protease and cleaved His_6_‐MBP were removed by passing the digestion reaction over a Co (II)‐charged TALON IMAC resin (Clontech). BorF was further purified using a Superdex 200 10/300 GL size exclusion column (GE Healthcare) in 20 mM Tris pH 7.5, 150 mM NaCl. BorF eluted from the size exclusion column in a single peak, with an elution volume of 78.2 mL (corresponding to a dimer).

BorF fractions are yellow in color, indicating copurification with flavin. To confirm the identity of the cofactor, purified BorF was denatured by boiling at 100°C for 5 min, followed by removal of the precipitated protein by centrifugation at 16 000 × *g* for 5 min. The yellow supernatant was analyzed by ESI‐MS using a Thermo Finnigan LCQ Deca XP MS System in negative ion mode. The major peak of *m/z* = 784.2 corresponds to the theoretical monoisotopic mass of the deprotonated molecular ion of FAD (784.1498).

BorF copurified with flavin (holo‐BorF) was used for crystallization because our target was the structure of BorF complexed FAD. The co‐purified FAD can be removed during purification by binding His_6_‐MBP‐BorF to an immobilized metal affinity column and washing with 2 M KBr and 2 M urea [41] to produce apo‐BorF, followed by tag cleavage and size exclusion as described above. Complete removal of flavin in the washing step was monitored with absorbance at 450 nm. Apo‐BorF was used for activity studies, so we could test the requirement of FAD for NADH oxidation by BorF (Figure 1B) and test the ability of BorF to reduce flavins other than FAD (Figure 1C).

Protein concentrations were measured using the Bradford assay (Bio‐Rad Laboratories). FAD concentration was measured using 450 nm absorbance and the molar extinction coefficient of FAD (11 300 M^−1^ cm^−1^). NADH concentrations were measured using 340 nm absorbance and the molar extinction coefficient for NADH (6220 M^−1^ cm^−1^). Protein purity was assessed by SDS‐PAGE analysis.

### 2.2 FR Activity

FR activity was determined by measuring depletion of NADH spectrophotometrically at 340 nm. Room temperature reactions were prepared containing 25 mM HEPES pH 7.5, 125–140 μM NADH, and either no enzyme, 0.2 μM apo‐BorF, or 0.2 μM apo‐BorF + 25 μM FAD in a total reaction volume of 200 μL. Absorbance measurements were recorded at 340 nm at room temperature using a Molecular Devices SpectraMax microplate reader and converted to concentrations using the extinction coefficient for NADH (6220 M^−1^ cm^−1^). To study substrate specificity, reactions were carried out with all six permutations of three hydride acceptors (25 μM FMN, riboflavin, or FAD), and two hydride donors (100 μM NADPH or NADH).

### 2.3 Crystallization

Purified holo‐BorF (10 mg/mL, 0.474 mM, in 20 mM Tris pH 7.5100 mM NaCl) was incubated with 1 mM FAD (MP Biomedical) for co‐crystallization of the BorF/FAD complex. Crystals were grown using the hanging drop vapor diffusion method, in which 1 μL of BorF/FAD solution was mixed with an equal volume of reservoir solution containing 0.1 M HEPES pH 7.6, 20% (*v/v*) polyethylene glycol (PEG) 400 and 5% (*v/v*) glycerol and equilibrated at room temperature against 200 μL of reservoir. Yellow crystals appeared after 2 days at room temperature and grew to overall dimensions of 0.03 × 0.03 × 0.025 mm^3^. Crystals were cryoprotected in mother liquor containing 25% (*v/v*) PEG 400 and 5% (*v/v*) glycerol, mounted and flash‐cooled in liquid N_2_.

### 2.4 Data Collection and Processing

Diffraction data were collected at a wavelength of 0.98757 Å and a temperature of 100 K using the oscillation method at the Life Sciences Collaborative Access Team beamline 21‐ID‐G at the Advanced Photon Source (Argonne National Laboratory). Reflections were indexed and integrated using *imosflm* [42, 43, 44], scaled and merged with *Scala* [45], and converted to structure factor amplitudes using *Truncate* [46]. The high‐resolution cutoff used for model building and refinement was 2.37 Å, reflecting the highest resolution shell with an average I/*σ*(I) > 2.0 (Table 1).

**TABLE 1:** Data collection and refinement data.

Diffraction source	APS beamline 21‐ID‐G
Wavelength (Å)	0.98757 Å
Temperature (K)	100
Detector	Marmosaic 300 mm CCD
Space group	*C*2
*a*, *b*, *c* (Å)	278.01, 62.34, 101.88
α, β, γ (°)	90, 110.84, 90
Resolution range (Å)	47.61–2.37 (2.45–2.37)
Total No. of reflections	306 720 (30373)
No. of unique reflections	66 699 (6590)
Completeness (%)	99.93 (99.97)
Redundancy	4.6 (4.6)
⟨*I*/*σ*(*I*)⟩	9.4 (2.0)
Overall *B* factor from Wilson plot (Å^2^)	44.21
*R*_merge_	0.100 (0.820)
*R*_meas_	0.113 (0.923)
CC_1/2_	0.995 (0.750)
Reflections used in refinement	66 699 (6590)
Reflections used for *R*_free_	1378 (129)
Final *R*_work_	0.215 (0.296)
Final *R*_free_	0.247 (0.351)
No. of non‐H atoms	
Protein	9688
Ligand (FAD)	424
Water	157
Total	10 269
R.M.S. deviations	
Bonds (Å)	0.004
Angles (°)	0.990
Clashscore	2.20
Average *B* factors (Å^2^)	
Protein	53.5
Ligand	52.6
Water	39.1
Ramachandran plot	
Most favored (%)	98.67
Allowed (%)	1.33

*Note:* Values for the outer shell are given in parentheses.

### 2.5 Structure Determination and Refinement

Molecular replacement was performed with *Phaser‐MR* [47] using chain A of PDB entry 4HX6 [33] (SgcE6; 42% sequence identity to BorF) as a search model. *Phenix* [48] was used for model building and refinement. Eight chains arranged as four homodimers were built within the asymmetric unit using the *Phenix* autobuild module [49]. After rigid body refinement using *Phenix.refine* [50], electron density for FAD was clearly visible for all eight subunits in *σ*A‐weighted *F*_*o*_ *–F*_*c*_ maps. The *LigandFit* [51, 52, 53] and *elBOW* [51] modules of *PHENIX* were used to fit FAD to the density. Iterative model building using *Coot* [54] and positional, real space, simulated annealing, and individual isotropic *B*‐factor refinement using *Phenix.refine* converged to a final model with an *R*_work_ of 21.9% and an *R*_free_ of 24.6%. Torsion‐based NCS restraints were used in the early rounds of refinement, and water picking and deletion was carried out automatically with *Phenix* followed by manual inspection of water density and hydrogen bonding using *Coot*. X‐ray/stereochemistry and X‐ray/ADP weights were optimized during the final round of refinement. Data collection and refinement statistics are presented in table 1. *MolProbity* [55] was used to validate the final model. DALI [56] was used to identify structural homologs. Figures were produced using Endscript/ESPript [57], PyMOL [58], and Ligplot^+^ [59]. Model coordinates and structure factors were deposited in the RCSB Protein Data Bank with PDB code 5CHO.

Prediction of potential BorF/BorH complexes was carried out using the pyDockWEB [60] and ClusPro [61] using coordinates from chains C and D of BorF/FAD (5CHO) and from chain C of BorH/FAD (8TTI) [14], and with AlphaFold3 [62] using the amino acid sequences of BorH and BorF. Geometric placement of chains in a possible BorF/BorH FAD‐bridged complex was done in PyMOL by treating BorH/FAD as a rigid body, orienting it to create a vector of approach between the adenosine of BorH/FAD and the adenosine of Chain C of BorF/FAD, and using a combination of translation and rotation about C5′ of FAD to find a position that minimized the distance between C5′ of BorH‐bound FAD and N5 of BorF‐bound FAD while minimizing steric clashes between the two proteins.

## 3 Results and Discussion

### 3.1 BorF Is a FR That Requires NADH and Prefers FAD

BorF was expressed in *E. coli* and purified by immobilized metal affinity chromatography, tag cleavage with Prescission protease, and size exclusion chromatography. After tag cleavage, BorF eluted from a HiLoad 16/60 Superdex 200 column in a single peak with an elution volume of 78.2 mL, corresponding to a dimer. A yellow color and absorbance at 450 nm in peak fractions indicated that BorF copurified with substoichiometric and variable amounts of flavin (holo‐BorF). To confirm the identity of the copurified flavin, a sample of purified holo‐BorF was denatured by boiling to release the flavin, which was analyzed by mass spectrometry and found to have *m/z* = 784.2, corresponding to the theoretical monoisotopic mass of the deprotonated molecular ion of FAD (784.15) and easily distinguishable from FMN (*m/z* = 455) and riboflavin (*m/z* = 375).

BorF oxidizes NADH to NAD^+^, as determined by NADH depletion monitored by absorbance at 340 nm. NADH oxidation requires the presence of both FAD and BorF (Figure 1B). BorF utilizes FAD as a substrate but is also able to reduce FMN and riboflavin; however, it is incapable of efficiently using NADPH as a hydride source (Figure 1C). Similar flavin promiscuity and specificity for NADH over NADPH has been reported for other short‐chain FRs in this family, including AbeF [15], PheA2 [63], HpaC_Tt_ [27], ActVB [64], StyB [65], SMOB [32], TftC [30], and ThdF [66].

### 3.2 Structure of the BorF/FAD Complex

The structure of the BorF/FAD complex was solved at 2.37 Å by molecular replacement using SgcE6 (4HX6‐A; 42% identity) as a search model. BorF co‐crystallized with FAD in space group *C*2 with four homodimers (chains AB, CD, EF, GH) per asymmetric unit. The crystallized construct comprises full‐length BorF (amino acid residues 1–196) with an additional N‐terminal proline remaining after affinity tag cleavage. No electron density was observed for the N‐terminal 30 amino acid residues, and 1–4 C‐terminal residues were absent from density for all chains. Backbone density for Chains A–E was clear and unbroken with well‐resolved side chains d except for several solvent‐exposed arginine side chains, which were truncated to Cβ. Chains F–H were built into noisier regions of the map that had weaker density for loop regions, resulting in higher temperature factors (average *B* factor for chains A–E = 44.1 Å^2^, average *B* factor for chains F–H = 73.4 Å^2^). The Cα R.M.S. deviations among the eight chains were 0.14–0.21 Å (Table S1). Our structural analysis will focus on the CD dimer, which has the greatest number of residues modeled and the lowest *B*‐factors. Electron density for FAD was visible in *σ*A‐weighted 2*F*_*o*_–*F*_*c*_ and *F*_*o*_–*F*_*c*_ maps in all eight chains, and it was modeled prior to refinement.

**FIGURE 2:**
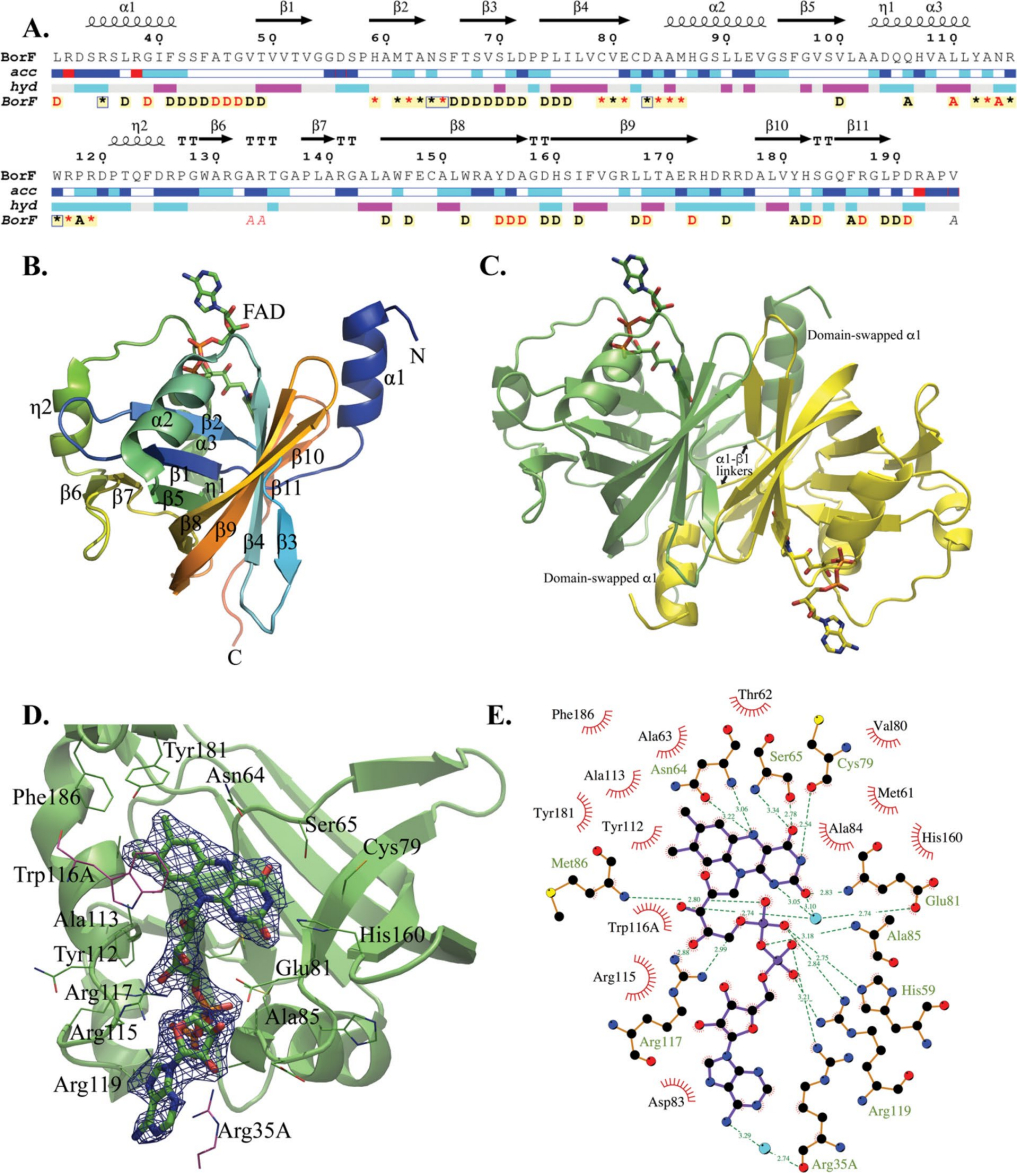
The X‐ray crystal structure of BorF/FAD. (A) BorF primary and secondary structure. Chain C of the model contains amino acids 31–195. Secondary structural elements are shown above the sequence. Relative solvent accessibility is shown below the sequence: White = buried (A < 0.1), cyan = intermediate (0.1 ≤ A ≤ 0.4), blue = accessible (0.4 ≤ A ≤ 1). Solvent accessibility was not calculated for Arg 32, Arg 28 and Arg 192, which were modeled without side chains (red boxes). Hydropathy is shown below the accessibility: Pink = hydrophobic, gray = intermediate and cyan = hydrophilic. Intermolecular contacts are displayed below hydropathy (Red = distance < 3.2 Å and black = distance = 3.2–5.0 Å). Residues contacting Chain A (in adjacent dimer in the ASU), Chain D (dimer partner) or FAD are denoted by A, D or *respectively. (B) Ribbon diagram of chain C of BorF/FAD with secondary structural elements labeled. FAD is shown as a stick model. (C) BorF/FAD homodimer (chain C in yellow, chain D in green), with FAD shown as a stick model. The domain‐swapped N‐terminal α‐helices and the α1–β1 linkers are labeled. (D) *F*_*o*_ *–F*_*c*_ Polder omit map (omitting FAD atoms) contoured at 3*σ* over final refined model of FAD from chain C (green stick model). Side chains contacting FAD in chain C are shown as green wire models. Side chains from chain A in adjacent dimer contacting FAD (Trp116A and Arg35A) are shown as magenta wire models. (E) Polar and nonpolar interactions between BorF and FAD. Hydrogen bonds are represented by dashed lines and hydrophobic interactions by spiked semicircles.

BorF (UNIPROT M9QXS1; GenBank AGI62216.1) belongs to a family of short‐chain oxidoreductases (InterPro IPR002563; Pfam PF01613; SMART SM00903) that includes other FR components of two‐component flavin‐dependent monooxygenases and halogenases, as well as a wider variety of confirmed and putative oxidoreductases, including enzymes that reduce cobalamin and iron. Members of this family are homodimers with protomers containing a split β‐barrel with Greek key topology, which has been described as a circular permutation of the ferredoxin reductase‐like flavin binding domain [67]. Each BorF subunit comprises 11 β‐strands, 3 α‐helices and two 3_10_ helices (Figure 2A,B). The N‐terminal helix α1 is domain‐swapped and sits atop the β‐barrel of its counterpart (Figure 2C). The split β‐barrel (β1–5 and β8–9) is capped on one end by helix α2 and by α1 from the dimer partner on the other. Packed against one face of the barrel is a subdomain inserted between β5 and β8 (η1, α3, η2, and a β6–β7 hairpin), along with loop β4–α2, that forms a groove that cradles the FAD ribityl, pyrophosphate and ribose groups. C‐terminal to the β‐barrel, a β10–β11 hairpin contributes to both the FAD binding site and the dimer interface.

The dimer interface (Figure 2C) buries approximately 2300 Å^2^ and involves 60 residues from each chain. The domain swapped N‐terminal helix α1 is amphipathic and inserts the side chains of Leu37C, Ile40C and Phe41C into the interior of the β‐barrel, where they pack against Leu71D, Ile76D, Val100D, Ala145D, and Phe147D (Figure S3A). The linkers connecting α1 to β1 in the two protomers (indicated by arrows in Figure 2C) are antiparallel to one another in extended conformations and interact via backbone hydrogen bonds (Figure S3B). One face of the β‐barrel (β3–β4–β9–β8) interacts with its counterpart in an antiparallel fashion mediated primarily by packing of hydrophobic side chains, a π‐stacking interaction (Tyr155C‐Tyr155D), and hydrogen bonds between the side chain of Tyr155C and the backbone of Asp156D and between the side chain of Asp159C and the amides of Leu71D and Asp72D (Figure S3C). Loop β2–β3 forms hydrophobic interactions and a hydrogen bond between Thr67C and Thr67D (at the N‐terminal ends of β3C and β3D). The β10–β11 hairpin forms an antiparallel β‐sandwich with its counterpart in the dimer partner with a hydrophobic core containing Val180 and Leu189 from both chains along with Cβ and Cγ of Arg187 and Cβ of His182. The side chain of Arg187C donates a hydrogen bond to Pro190D and forms a salt bridge with Asp191D, and the side chain of Arg172C forms a hydrogen bond with the side chain of Ser183D (Figure S3D).

### 3.3 The FAD Binding Site

FAD was modeled into *σ*A‐weighted 2*F*_*o*_–*F*_*c*_ and *F*_*o*_–*F*_*c*_ maps in all eight chains (final refined model with a *F*_*o*_–*F*_*c*_ Polder omit map omitting the FAD is shown in Figure 2D). The binding of the isoalloxazine is consistent with previous crystal structures of short chain FRs bound to FAD [26, 30, 32, 33] or FMN [27, 34, 68]. The isoalloxazine occupies a pocket surrounded by strand β4, loop β2–β3, loop α1–β1, helix α3, and the β10–β11 hairpin. The 2,4‐pyrimidinedione portion of the isoalloxazine forms hydrogen bonds with backbone atoms of Asn64, Ser65, Cys79, and Glu81, the side chain of Ser65, and a water molecule that also forms hydrogen bonds to the side chain of Glu81 and O3 of the ribityl group of FAD (Figure 2E). The backbone amide of Asn64 forms a hydrogen bond to FAD N5. The dimethylbenzene binds between helix α3 and the β10–β11 hairpin and contacts the side chains of Val48, Thr62, Asn64, Tyr181, Phe186, and Ala113 (Figure 2D,E). Trp116 from an adjacent dimer in the asymmetric unit makes a hydrophobic crystal contact with the dimethylbenzene portion of the FAD (Figure 2D,E).

The ribityl group of FAD binds in an extended conformation along strand β2, hydrogen bonding with the backbones of Thr62, Tyr112, and Ala113, a water molecule, and the side chain of Arg117. The pyrophosphoryl group extends at a right angle to the ribityl group and interacts via salt bridges with the side chains of His59, Arg117, and Arg119 as well as Arg35 from an adjacent dimer in the asymmetric unit, and via hydrogen bonds with the backbones of Ala85 and Met86 (Figure 2D,E). The adenosine emerges from the groove at the interface between dimers in the asymmetric unit. The ribose forms hydrogen bonds with the side chains of Glu81 and Arg115. As a result of crystal packing, a cation‐π interaction is formed between Arg35 from chain A (in an adjacent dimer in the asymmetric unit) and the FAD adenine group of chain C, which is approximately 4 Å away from and oriented parallel to the guanidinium group of the Arg35A side chain. The Arg35A side chain also forms a salt bridge with one of the phosphoryl groups of FAD as noted above.

### 3.4 Comparison of BorF to Other Short Chain FRs

**FIGURE 3:**
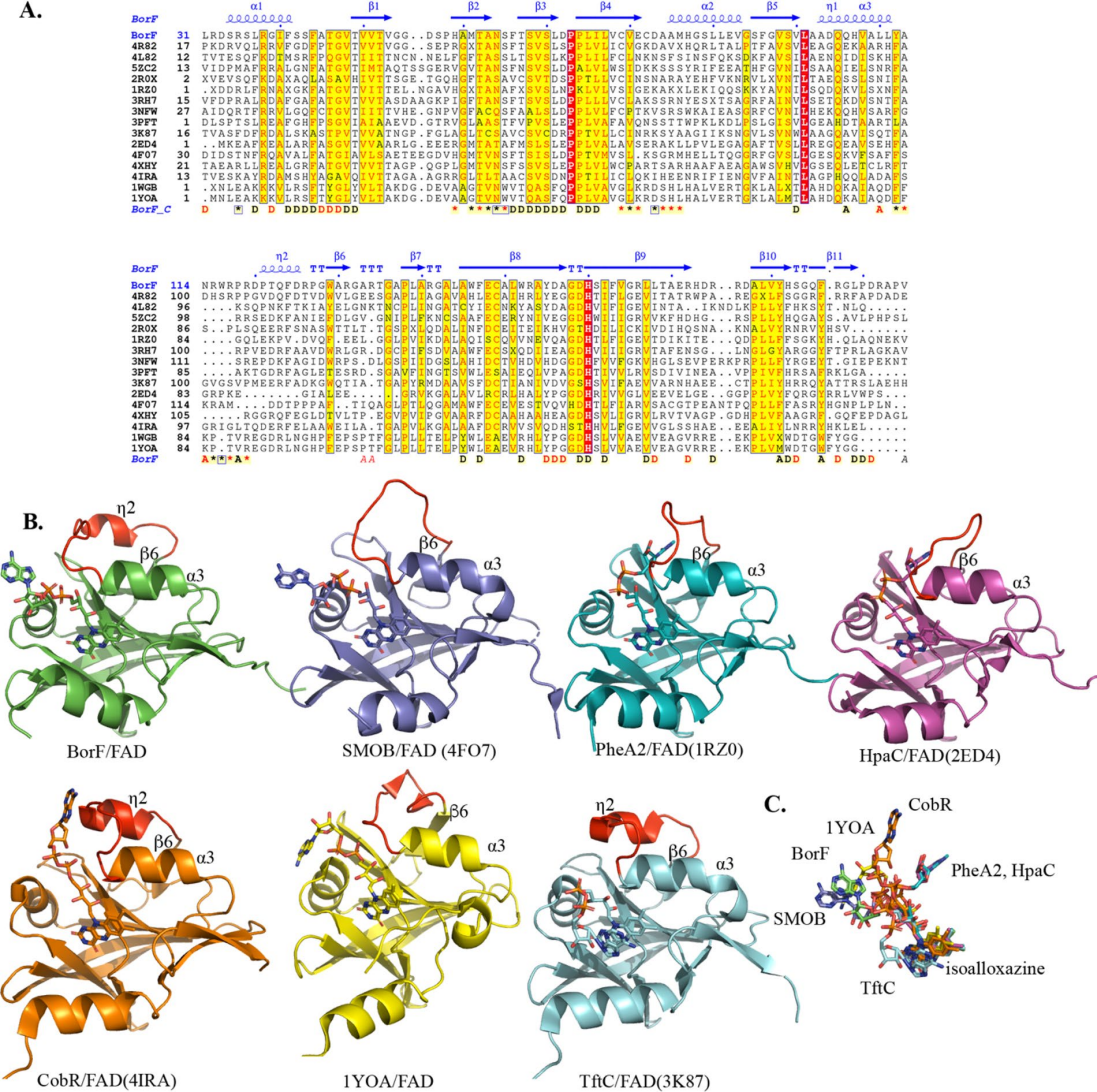
Structural homologs of BorF. (A) Multiple sequence alignment of BorF with structural homologs (see Table S2). BorF secondary structural elements are indicated above the alignment. BorF residues contacting other subunits or FAD are labeled as in Figure 2A. Strictly conserved residues have a red background. (B) The core β‐barrel of BorF superimposes well with select structural homologs, but the region between α3 and β6 (residues 112–129 in BorF, shown in red), which forms part of the FAD binding site, varies substantially in length and sequence and contributes to the different conformations observed for the adenosine portion of FAD. (C) Superposition of FAD from BorF and homolog complexes in the same orientation as (B).

DALI was used to identify structural homologs to chain C of BorF (Figure 3A and Table S2). PDB entries representing 30 different proteins with DALI Z‐scores of 19.9 or higher had Cα RMSD values between 1.4 and 2.5 Å with BorF chain C. All are known or putative flavin‐binding proteins from bacteria or archaea and can be divided into three categories. The first are enzymes known to be reductase components of two‐component flavin‐dependent monooxygenase systems, including SgcE6 from *Streptomyces globisporus* C‐1027 (4R82/4HX6) [33], C1‐HPAH from *Acinetobacter baumannii* (5ZC2) [34], PheA2 from *Geobacillus thermoglucosidasius* (1RZ0/1RZ1) [26], TftC from *Burkholderia cepacian* (3K86/3K87/3K88) [30], HpaC from *Thermus thermophilus* (2ED4/2ECU/2ECR) [28], HpaC from *Sulfolobus tokodaii* (2D36/2D37/2D38) [27], and SMOB from *Pseudomonas* sp. *Y2* (4F07) [32]. The second category are enzymes which use reduced FMN to reduce a variety of chemically distinct substrates, such as corrin reductase CobR (3CB0/4IRA) from *Brucella melitensis* [29], ferric reductase FerA from *Paracoccus denitrificans* (4XJ2) [68], ketoreductase PlmKR1 from *Streptomyces* sp. HK803 (4HXY) [69, 70], ferric reductase FeR from *Archaeoglobus fulgidus* (1I0S/1I0R), YP_005476 from *Thermus thermophilus* (3ZOE, 3ZOF, 3ZOH) [71], and 2‐nitrobenzoate nitroreductase NbaA from *Pseudomonas fluorescens* (4Z85) [72]. The third set of homologs are putative flavin‐binding proteins of unknown function solved by structural genomics projects, including 4L82, 2R0X, and 3RH7.

The core β‐barrel of BorF superimposes well with its structural homologs (Figure 3B), but the region between α3 and β5 (residues 112–129 in BorF), which forms part of the FAD binding site, varies substantially in length and sequence (Figure 3B). The binding of the isoalloxazine portion of FAD, which primarily involves contacts with main chain atoms, is well‐conserved between the BorF/FAD complex and structures of FAD and FMN bound to structural homologs of BorF. However, in structures with bound FAD, the ribityl, pyrophosphoryl, and adenylyl groups are found in a range of different conformations, primarily due to structural variation in the region between α3 and β6 (Figure 3B,C). In BorF, α3 is followed by loop α3–η2 (residues 112 to 120), which forms hydrogen bonds and salt bridges using backbone and side chain atoms to the ribityl, pyrophosphate, and adenosine of FAD. Residues 121–125 make up 3_10_ helix η2, which is roughly antiparallel to α3, and a loop from 126 to 129 connects to the N‐terminus of the β6–β7 hairpin. Many proteins in this structural family preferentially bind FMN rather than FAD [8, 36]. The higher conservation of the binding sites and greater number of interactions for the isoalloxazine and ribityl groups compared to the AMP moiety of FAD suggest that the fold may have originally evolved as an FMN‐binding module. Co‐evolution of some short chain FRs with FAD‐specific monooxygenases and halogenases may have selected for residues that make additional interactions with the AMP.

### 3.5 Trp116 From an Adjacent BorF Dimer Obstructs the Nicotinamide Binding Site

Some homologs of BorF have had structures determined of dead‐end ternary complexes with FAD and NAD^+^ (4R82 [33], 1RZ1 [26], 2ED4 [28], 2D37 [27], 3K88 [30]). In these structures, the NAD^+^ binds in a folded conformation in which the adenine stacks on the nicotinamide ring, which in turn stacks on the central ring of the isoalloxazine group of FAD, juxtaposing C4 of the nicotinamide with N5 of the FAD for hydride transfer (Figure S2A). We collected data on multiple BorF crystals grown in the presence of both FAD and NAD^+^ or grown in the presence of FAD and soaked with NAD^+^ but could not detect any electron density in the substrate binding site that would indicate the presence of the dead end BorF/FAD/NAD^+^ ternary complex. Crystal packing explains the lack of observed NAD^+^ binding, as the side chain of Trp116A is occupying the position where the ribose of NAD^+^ and two water molecules bridging the NAD^+^ and PheA2 are found in the PheA2 ternary complex (Figure S2B).

### 3.6 The Adenosine Moiety of FAD Is Solvent‐Exposed and Adopts Variable Conformations in Different FRs

Of the crystal structures of BorF homologs with FAD bound, the orientation of the FAD pyrophosphoryl group and adenosine in styrene monooxygenase component 2 (SMOB/FAD; 4F07, Figures 3B and S4A) is most like BorF/FAD. Compared to BorF, SMOB has a shorter insertion between α3 and β6 and lacks the 3_10_ helix η2. A second conformation of FAD, strikingly different from that seen in BorF and SMOB, can be seen in PheA2 (1RZ0, Figures 3B and S4B) and HpaC_Tt_ (2ED4, Figures 3B and S4C). When comparing BorF/FAD to PheA2/FAD, the isoalloxazine, ribityl and first phosphoryl group superimpose well, but the second phosphoryl group in PheA2 is rotated by 180°, directing the adenosine away from the dimer interface and instead into the space between β2, α3, and loop α3–β6, where the adenine is stabilized by 2 H‐bonds and a π‐stacking interaction. This orientation of the adenosine is not possible in BorF, as loop α3–η2 occupies the space occupied by the adenine in PheA2/FAD. The region between α3 and β6 is shorter in PheA2 (12 amino acids compared to 17 in BorF) and lacks the 3_10_ helix η2. HpaC_Tt_ also lacks the 3_10_ helix, has a shorter β5–β6 hairpin, and has a nearly identical FAD conformation to PheA2.

Several other FRs have structural differences that sterically prevent the FAD from adopting the conformation found in the BorF structure. In the corrin reductase CobR (4IRA, Figures 3B and S4D), the isoalloxazine and C1′–C3′ of the ribose align with the FAD conformation found in BorF, but the rest of the FAD is shifted significantly due to bulkier residues in loop β4–α2 and the first turn of α2 (particularly His67 and Glu69, which are both Ala in BorF) on one side, and a different backbone conformation for loop α3–η2 that moves η2 closer to α3. Putative flavoprotein 1YOA (Figures 3B and S4E) also obstructs BorF's adenine conformation with bulkier residues in loop β4–α2, and changes in the α3–β6 region including the deletion of η2 accommodate an alternate positioning of the pyrophosphate and ribose. FAD bound to TftC adopts two different conformations. In the ternary TftC/FAD/NAD^+^ complex (3K88), the adenine binds in an extended conformation that clashes with loop α3–η2 in BorF, but in the TftC/FAD binary complex (3K87), one protomer has the extended FAD conformation, and the other has a folded FAD with the adenine stacked with the isoalloxazine in the position occupied by the nicotinamide of NAD^+^ in the ternary complex (Figures 3B and S4F).

### 3.7 Comparison of BorF/FAD and BorH/FAD Suggest That a Bridged Flavin Complex Is Unlikely

Most two‐component systems rely on freely diffusing flavins, but a few exhibit transient or weak protein–protein interactions [36]. Consistent with this, size‐exclusion chromatography of BorF/BorH results in separate peaks with their individual retention times, indicating they do not form a stable complex on the SEC timescale, although transient or weak interactions cannot be ruled out. Rigid‐body docking of the BorF CD dimer with BorH (8TTI chain C [14]) using pyDockWEB [60] and ClusPro [61] predicted only weakly interacting complexes without the FAD binding sites of the two proteins near the interface. AlphaFold3 [62] prediction of a BorF/BorH complex with FAD bound produced a top result with a low interface predicted template modeling score (ipTM = 0.37), indicating an unreliable prediction, though in this model the FAD‐binding sites of both proteins are located near the interface. These results argue against the formation of a stable BorF/BorH complex.

In BorF, the isoalloxazine of the FAD is enclosed within the active site, and the adenosine group is solvent exposed (Figure S5A). In SMOB, it has been suggested that the protrusion of the adenosine from the FR may allow it to serve a bridging function between the FR and the monooxygenase [32, 73]. In our crystal structure of BorH/FAD [14], the FAD is buried in a cleft (Figure S5B), and the isoalloxazine and ribityl groups have much weaker electron density than the adenosine group, suggesting mobility of the isoalloxazine while the adenosine group remains in place. This was previously reported for the tryptophan‐6‐halogenase Thal, leading to the hypothesis that rather than releasing the flavin into solution, the adenosine may stay bound to the Thal as the isoalloxazine moves back and forth between the FDH and the FR [74], without requiring a stable protein–protein complex.

The maximum distance between C5′ of the adenosine and N6 of the isoalloxazine in a fully‐extended FAD is approximately 15 Å. For the adenosine to stay bound to BorH while the isoalloxazine binds to the active site of BorF, the two proteins would need to approach at least transiently so that the distance between N6 of the BorF‐bound isoalloxazine is within ~15 Å of C5′ of the BorH‐bound adenosine. To use our crystal structures to assess if this is plausible, we performed a rigid body geometric placement of BorH/FAD relative to BorF/FAD, using rotation and translation to minimize the distance between these atoms while avoiding steric clashes (Figure S5C). The resulting placement gave a distance of ~27 Å between these atoms, suggesting that a FAD‐bridged interaction between BorF and BorH is unlikely, though conformational flexibility could reduce this distance. The α3–β6 region previously discussed forms part of the BorH interface, suggesting that this variable structural feature influencing FAD conformation could play a role in flavin handoff or subunit selectivity. Even without formation of a stable protein–protein complex or a flavin‐bridged complex, the ability of the two flavin binding sites to approach without steric clashes could permit some channeling or shielding of reduced flavin to occur.

## 4 Conclusion

The crystal structure of the thermostable FR BorF reveals a homodimer with the characteristic short‐chain FR fold, featuring a conserved isoalloxazine binding pocket and an adenosine conformation influenced by the conformation of α3–β6 region. Structural variation in this region contributes to diverse FAD binding modes observed across members of this family. Crystal packing explains why we were unsuccessful in capturing a ternary complex with NAD^+^. Structural comparisons with BorH, along with rigid body docking analyses, suggest that it is unlikely FAD can bridge the FAD and FR simultaneously.

The BorH and BorF crystal structures provide a foundation for investigating the structural basis of their thermostability and may guide efforts to engineer more thermostable FR/FDH systems for biocatalysis. The structural variability of the α3–β6 region and its influence on adenosine positioning identifies a promising target for protein engineering aimed at tuning flavin affinity or improving flavin transfer efficiency. The experimental models of BorF and BorH offer a starting point for more rigorous computational modeling to investigate transient complex formation or other mechanisms of flavin transfer.

## Author Contributions

Z.M. carried out expression and purification of BorF, crystallization, X‐ray diffraction data collection, molecular replacement, model building, refinement, and structural analysis. E.W.R. performed BorF expression, purification, and crystallization. A.J.S. carried out expression and purification of BorF, and flavin reductase activity assays. J.J.B. carried out model building and refinement, data analysis, and structural analysis. The manuscript was written by Z.M. and J.J.B.

## Conflicts of Interest

The authors declare no conflicts of interest.

## Supporting information


**Figure S1:** Proposed mechanisms of FR (flavin reductase) and FDH (flavin‐dependent halogenase).
**Figure S2:** BorF crystal contact obstructs NAD+ binding site.
**Figure S3:** The BorF dimer interface.
**Figure S4:** Diversity of FAD binding conformations in BorF and structural homologs.
**Figure S5:** BorF cannot reduce FAD that is still bound to BorH.
**Table S1:** Pairwise backbone comparisons of BorF chains to Chain C.
**Table S2:** Structural homologs of BorF from the PDB.
**Table S3:** Nucleotide and amino acid sequences used in this study.

## Data Availability

The data that support the findings of this study are openly available in Protein Data Bank at https://www.rcsb.org/structure/5CHO, reference number 5CHO.
