# Supplementary material for: The X‐Ray Crystal Structure of BorF, the Flavin Reductase Subunit of a Two‐Component Flavin‐Dependent Tryptophan Halogenase
Source: Proteins. 2026 Mar 18;94(8):1529–40. doi: 10.1002/prot.70131 (PMC13327496; doi:10.1002/prot.70131)
Supplement: Supplementary file 1 — Figure S1: Proposed mechanisms of FR (flavin reductase) and FDH (flavin‐dependent halogenase). Figure S2: BorF crystal contact obstructs NAD+ binding site. Figure S3: The BorF dimer interface. Figure S4: Diversity of FAD binding conformations in BorF and structural homologs. Figure S5: BorF cannot reduce FAD that is still bound to BorH. Table S1: Pairwise backbone comparisons of BorF chains to Chain C. Table S2: Structural homologs of BorF from the PDB. Table S3: Nucleotide and amino acid sequences used in this study. [file PROT-94-1529-s001.pdf]

SUPPLEMENTARY MATERIAL

The X-ray crystal structure of BorF, the flavin reductase subunit of a two-component flavin-dependent tryptophan halogenase

Zheng Ma, Emily W. Rady, Aravinda J. de Silva and John J. Bellizzi III

Figure S1: Proposed mechanisms of FR (flavin reductase) and FDH (flavin-dependent halogenase).

Figure S2: BorF crystal contact obstructs NAD^+ binding site.

Figure S3. The BorF dimer interface.

Figure S4: Diversity of FAD binding conformations in BorF and structural homologs.

Figure S5: BorF cannot reduce FAD that is still bound to BorH.

Table S1. Pairwise backbone comparisons of BorF chains to Chain C

Table S2. Structural homologs of BorF from the PDB

Table S3. Nucleotide and amino acid sequences used in this study.

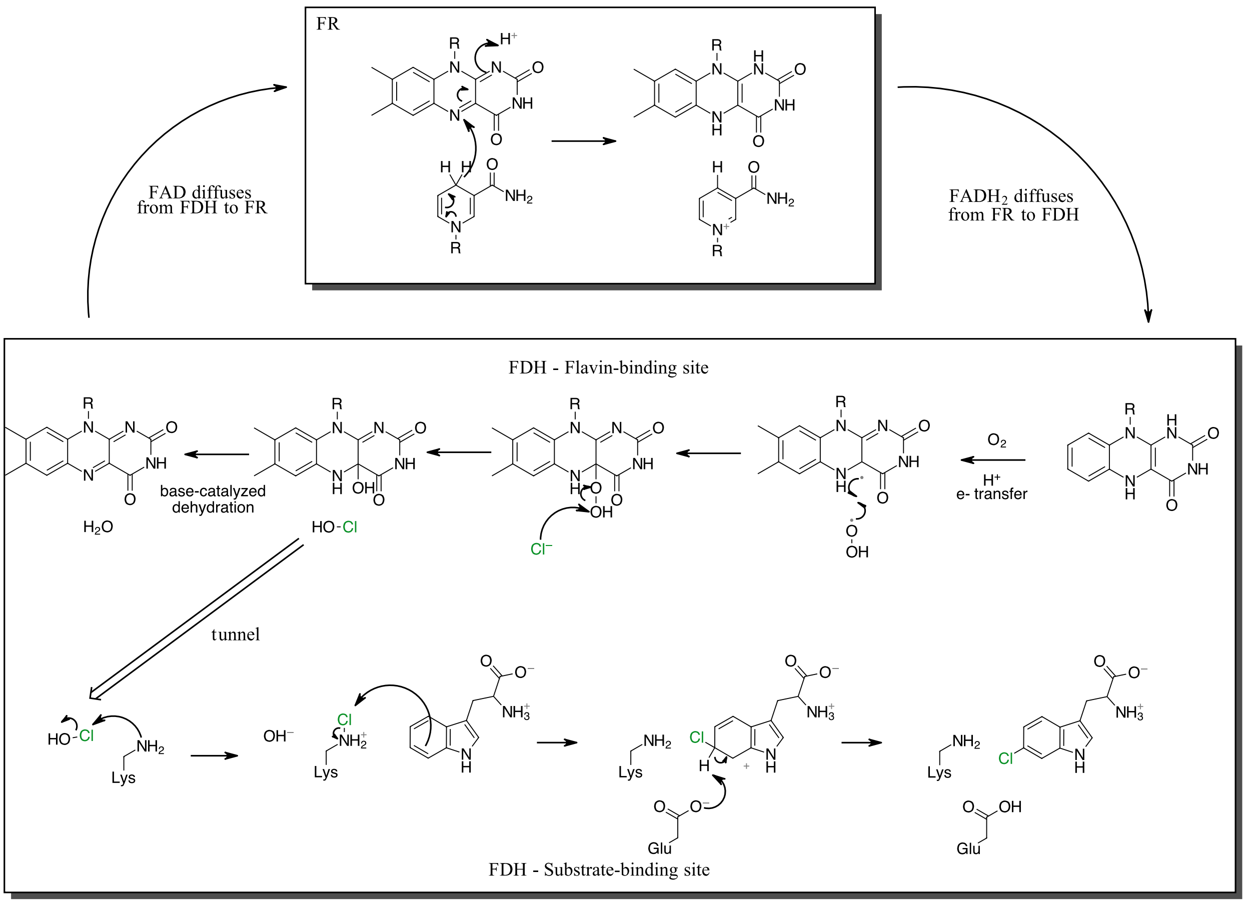

Figure S1: Proposed mechanisms of FR (flavin reductase) and FDH (flavin-dependent halogenase). The FR uses hydride transfer from NADH to reduce FAD to $FADH_2$, which diffuses to the flavin-binding site of the FDH. The FDH uses $FADH_2$, O_2, and Cl^- to form HOCl, which diffuses down a tunnel to the substrate binding site, where a catalytic Lys and Glu facilitate electrophilic aromatic substitution of an aromatic substrate[1].

A.

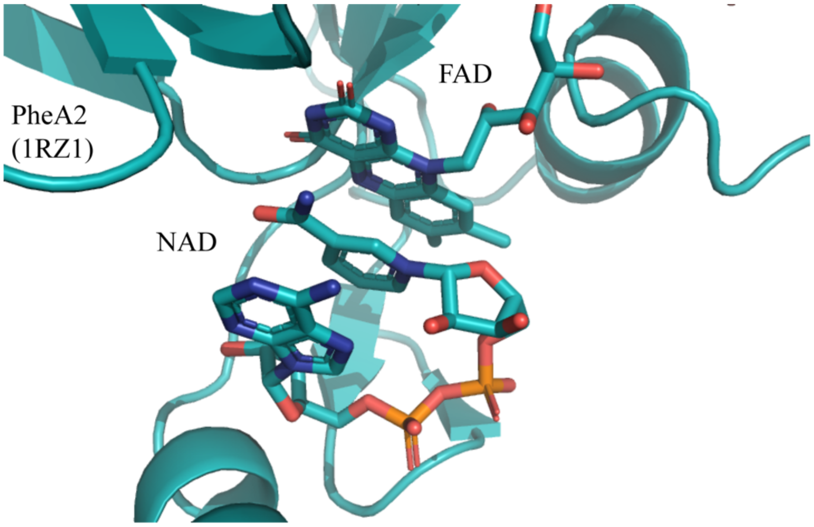

B.

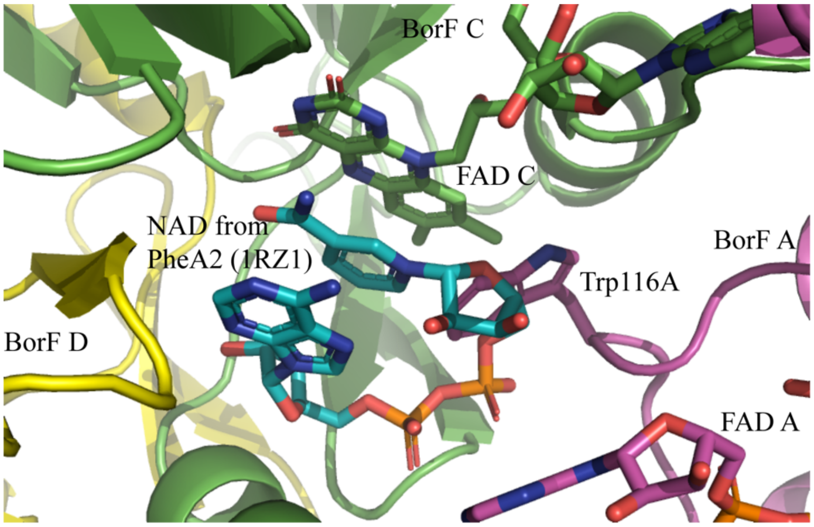

Figure S2: BorF crystal contact obstructs NAD^+ binding site.

A. The crystal structure of PheA2/FAD/NAD^+ structure (1RZ1 chain A, turquoise[2]) shows the NAD^+ in a folded conformation, with the nicotinamide ring sandwiched between the adenosine and FAD's isoalloxazine.

B. NAD^+ (turquoise) from chain A of the PheA2/FAD/NAD^+ structure (1RZ1) is shown in the active site of chain C of BorF/FAD (5CHO, green) after superimposing the two chains. A crystal contact from BorF chain A (in an adjacent dimer in the asymmetric unit; magenta) obstructs the NAD^+ binding site in BorF chain C (green). The side chain of Trp116A is occupying the position where the ribose of NAD^+ and two water molecules bridging the NAD^+ and PheA2 are found in the PheA2 ternary complex The side chain of Trp116 adenine of chain A blocks the position where the nicotinamide-connected ribose binds in the PheA2 ternary complex structure. This crystal contact would block NAD^+ binding and explains why BorF/FAD crystals grown in the presence of NAD^+ or soaked in NAD^+ had no electron density for NAD^+.

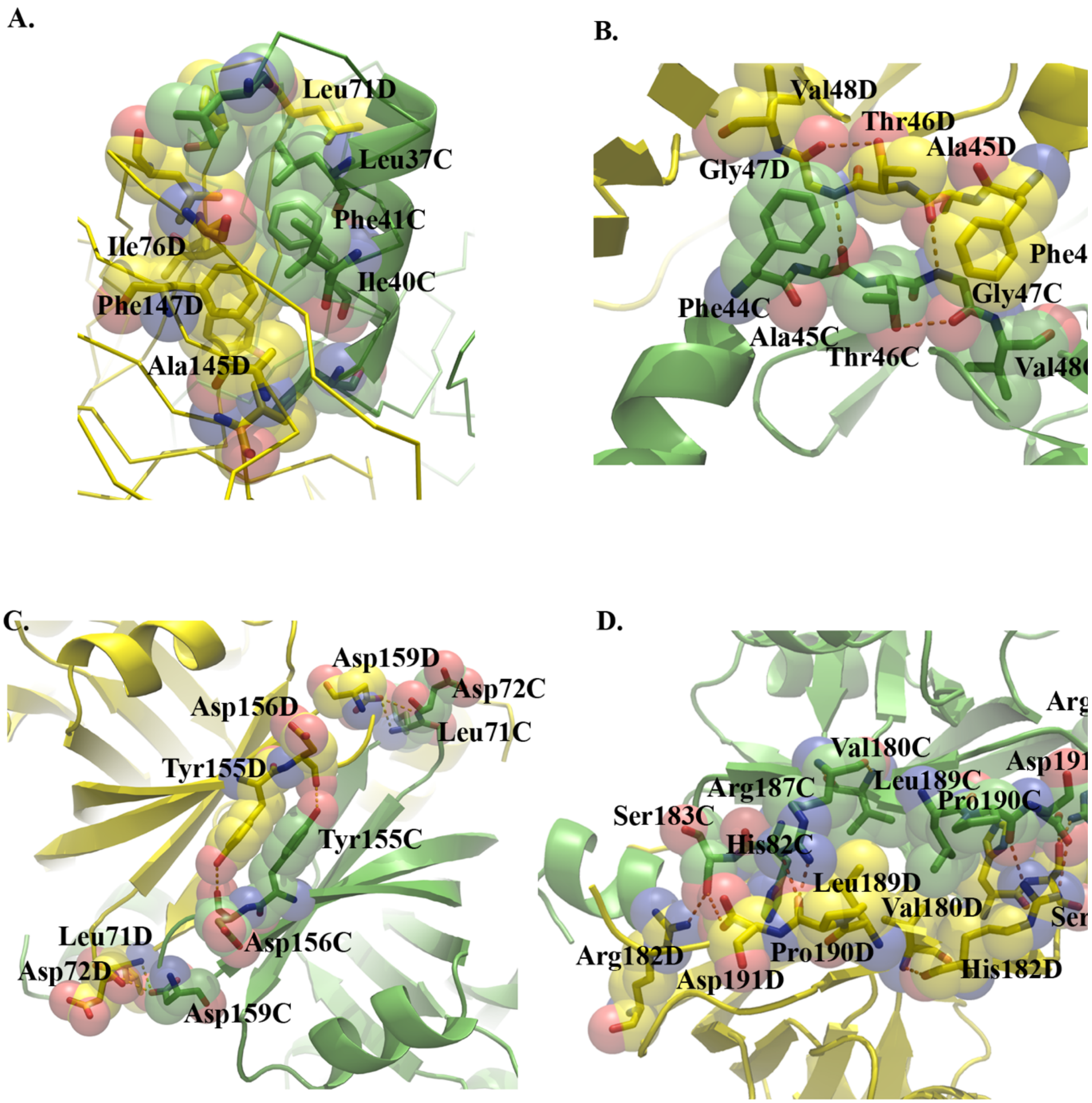

Figure S3. The BorF Dimer Interface

A. Helix α1 of chain C is domain-swapped and inserted into the β-barrel of chain D.
B. The extended linkers connecting α1 to β1 pack together in an antiparallel fashion.
C. The β-barrels form an interface centered around π-stacking of Tyr155C and Tyr155D.
D. The β10-β11 hairpin forms an antiparallel β-sandwich with its counterpart.

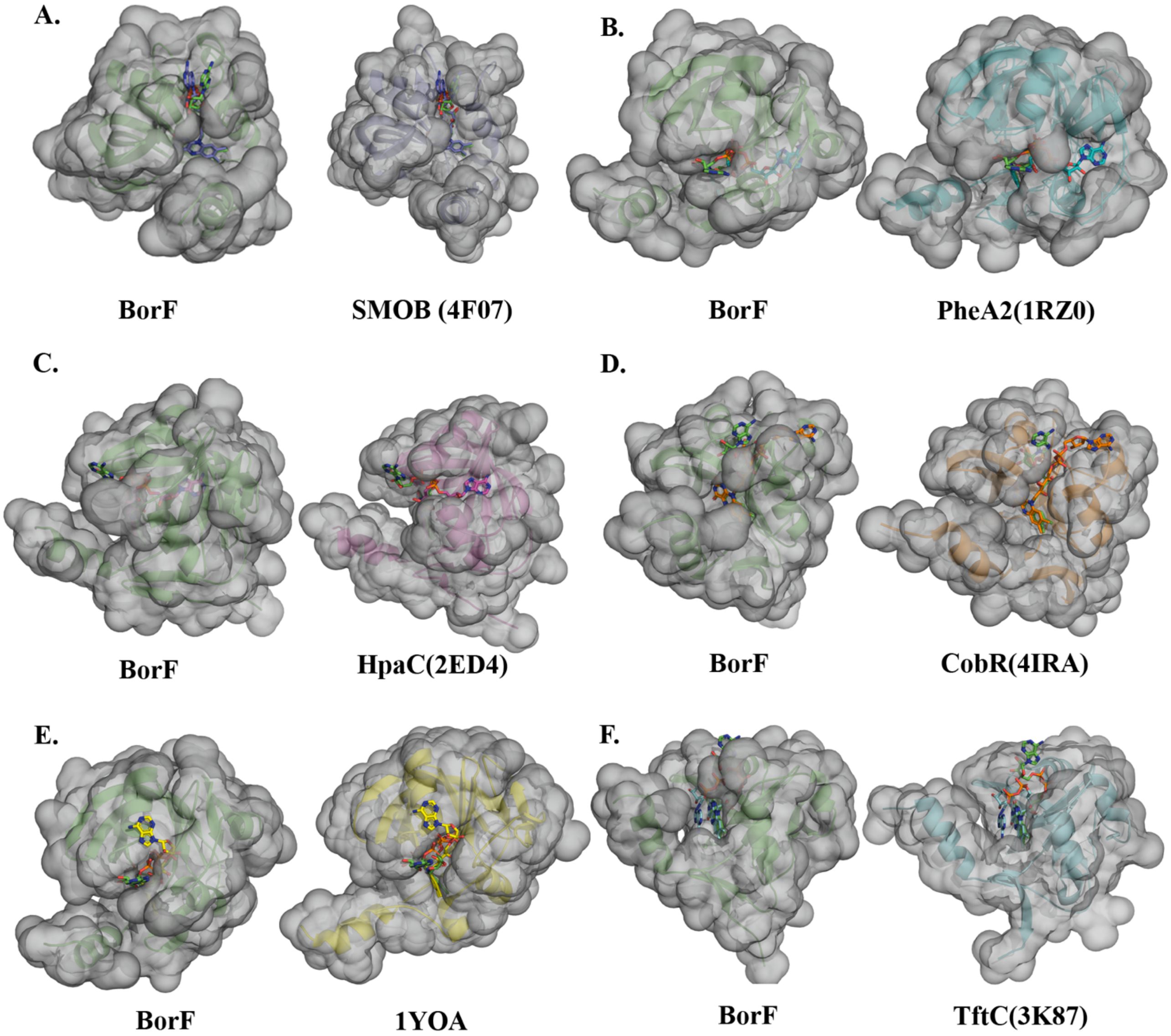

Figure S4: Diversity of FAD binding conformations in BorF and structural homologs.

In panels A-F, the left image shows the surface of the BorF/FAD complex with FAD from the homolog complex superimposed, and the right images shows the surface of the homolog/FAD complex with the FAD from BorF superimposed. Both images in each panel are in the same orientation as one another, but the orientation differs from panel to panel to show differences more clearly. **A.** BorF (green) and SMOB[3] (4F07, purple). **B.** BorF and PheA2[2] (1RZ0, turquoise) **C.** BorF and $HpaC_{Tt}$[4] (2ED4, magenta). **D.** BorF and CobR[5] (4IRA, orange) **E.** BorF and 1YOA (yellow) **F.** BorF and TftC[6] (3K87, cyan).

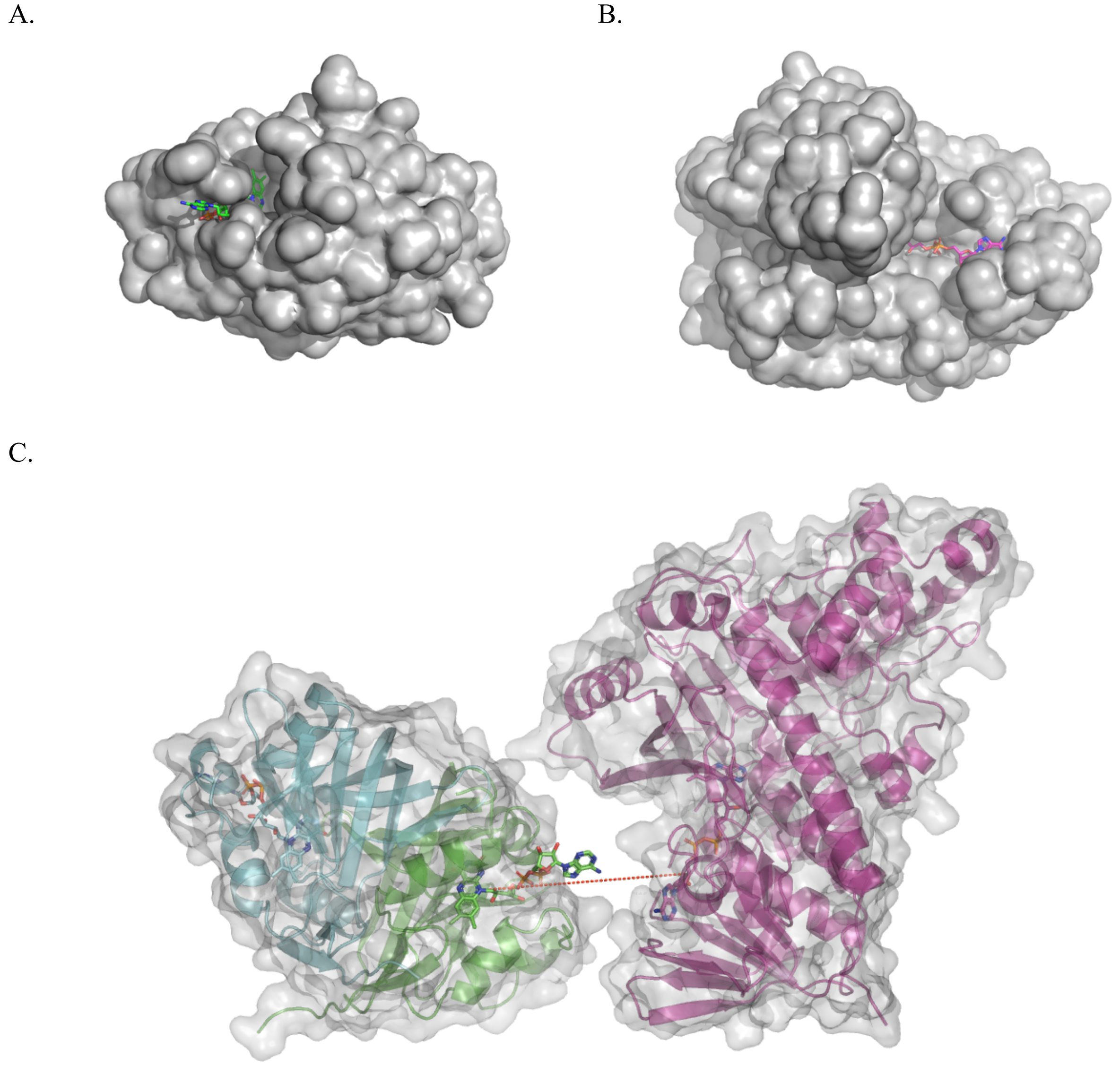

Figure S5: BorF cannot approach BorH close enough for FAD to bridge both subunits without conformational change.

A. The adenosine in BorF/FAD protrudes from the surface and is solvent-exposed

B. The adenosine in BorH/FAD is bound within a crevice on the surface.

C. Rigid body placement of BorF/FAD (5CHO chain C cyan, Chain D green) and BorH/FAD[7] (8TTI chain C, magenta). The red dashed line connects C5' of the adenosine from the BorH-bound FAD and N6 of the isoalloxazine from the BorF-bound FAD. The maximum distance between those two atoms in a fully extended conformation of FAD is 15 Å, and the distance shown in the figure is 27 Å. This indicates that BorF is not capable of reducing the isoalloxazine of an FAD molecule still bound to BorH via its adenosine without conformational change in one or both proteins.

Table S1. Pairwise backbone comparisons of BorF chains to Chain C

Chain	Residues	Residues aligned	Cα rmsd
A	30-194	165	0.201
B	31-193	163	0.201
D	31-194	164	0.169
E	33-193	161	0.212
F	33-192	160	0.142
G	33-192	160	0.185
H	34-193	160	0.161

Table S2. Structural homologs of BorF from the PDB

PDB	Complex	UniProt	Protein	Gene name	Organism	DALI Z-score	Cα rmsd	Residues aligned	% sequence Identity
4r82	FAD/NAD	Q8GME2	SgcE6 Flavin reductase	SGCE6	*Streptomyces globisporus*	25.1	1.4	156	48
4l82	FMN	Q4UKE8	Uncharacterized protein	RF_1132	*Rickettsia felis*	25.2	1.5	155	32
4hx6	apo	Q8GME2	SgcE6 Flavin reductase	SGCE6	*Streptomyces globisporus*	25.1	1.5	158	48
5zc2	FMN	Q6Q271	p-hydroxyphenylacetate 3-hydroxylase, reductase component	C1-hpah	*Acinetobacter baumannii*	23.1	1.6	154	32
5zyr	FMN	Q6Q271	p-hydroxyphenylacetate 3-hydroxylase, reductase component	C1-hpah	*Acinetobacter baumannii*	20.3	1.6	154	33
2r0x	apo	Q0I3S1	Possible flavin: NADH reductase	ycdH HS_1225	*Haemophilus somnus*	24	1.7	155	23
1rz0	FAD	Q9LAG2	PheA2 phenol 2-hydroxylase component B	pheA2	*Geobacillus thermoglucosidasius*	23	1.7	149	31
1rz1	FAD/NAD	Q9LAG2	PheA2 phenol 2-hydroxylase component B	pheA2	*Geobacillus thermoglucosidasius*	22.9	1.7	149	31
3rh7	FMN	Q92ZM6	Uncharacterized protein	SMa0793	*Rhizobium meliloti*	22.1	1.7	154	40
3nfw	apo	E5Q9D7	nitrilotriacetate monooxygenase component B	NTA-Mo	*Mycolicibacterium thermoresistibile*	21.7	1.7	156	29
3pft	FMN	B6CDL6	DszD Flavin reductase (Oxidoreductase)	AFA91_09010	*Mycobacterium goodii*	21.7	1.7	152	28
3k86	apo	O87008	TftC chlorophenol-4-monooxygenase component 1	tftC	*Burkholderia cepacia*	23.4	1.8	158	27
3k88	FAD/NADH	O87008	TftC chlorophenol-4-monooxygenase component 1	tftC	*Burkholderia cepacia*	23.3	1.8	158	27
3k87	FAD	O87008	TftC chlorophenol-4-monooxygenase component 1	tftC	*Burkholderia cepacia*	23.5	1.9	159	26
2ed4	FAD/NAD	Q5SJP7	HpaC 4-hydroxyphenylacetate 3-monooxygenase, reductase component	TTHA0961	*Thermus thermophilus (strain HB8)*	21.8	1.9	145	33
2ecu	apo	Q5SJP7	HpaC 4-hydroxyphenylacetate 3-monooxygenase, reductase component	TTHA0961	*Thermus thermophilus (strain HB8)*	21.7	1.9	145	33
2ecr	apo	Q5SJP7	HpaC 4-hydroxyphenylacetate 3-monooxygenase, reductase component	TTHA0961	*Thermus thermophilus (strain HB8)*	21.7	1.9	145	33
4f07	FAD	O33495	Styrene monooxygenase component 2	styB	*Pseudomonas sp. Y2*	21.5	1.9	146	32
4xj2	FMN	A1B5I2	FerA Flavin reductase domain protein, FMN-binding protein	Pden_2689	*Paracoccus denitrificans*	21.2	1.9	153	38
4xhy	apo	A1B5I2	Flavin reductase domain protein, FMN-binding protein	Pden_2689	*Paracoccus denitrificans*	21.1	1.9	153	38
3cb0	FMN	Q8YHT7	CobR corrin reductase	BMEI0709	*Brucella melitensis*	23.2	2	159	29
4ira	FAD	Q8YHT7	CobR corrin reductase	BMEI0709	*Brucella melitensis*	23.2	2	159	29
2qck	apo	A0JVA7	Flavin reductase domain protein, FMN-binding protein	Arth_1583	*Arthrobacter sp. (strain FB24)*	21.6	2	152	22
2d37	FMN/NAD	Q974C9	HpaC Putative phenol hydroxylase small component	pheA2	*Sulfolobus tokodaii*	21.4	2	151	26
2d38	FMN: NADPH	Q974C9	HpaC Putative phenol hydroxylase small component	pheA2	*Sulfolobus tokodaii*	21.2	2	151	26
2d36	FMN	Q974C9	HpaC Putative phenol hydroxylase small component	pheA2	*Sulfolobus tokodaii*	20.8	2.1	152	26
1wgb	apo	Q72LK7	Probable flavoprotein	TT_C0052	*Thermus thermophilus*	20	2.5	151	24
1yoa	FAD/FMN	Q5SL73	Probable flavoprotein	TTHA0420	*Thermus thermophilus HB8*	19.9	2.5	151	24

Table S3. Nucleotide and amino acid sequences used in this study.

BorF amino acid sequence (Uniprot M9QXS1).	MEGSVNGSQRGNGSQRERVPEPGAGPTTDL<br>LRDSRSLRGIFSSFATGVTVVTVGGDSPHA<br>MTANSFTSVSLDPPLILVCVECDAAMHGSL<br>LEVGSFGVSVLAADQQHVALLYANRWRPRD<br>PTQFDRPGWARGARTGAPLARGALAWFECA<br>LWRAYDAGDHSIFVGRLLTAERHDRRDALV<br>YHSGQFRGLPDRAPVE
borF codon-optimized nucleotide sequence (GenBank MW847680)	ATGGAAGGTAGCGTTAATGGTAGCCAGCGT<br>GGTAATGGTTCACAGCGTGAACGTGTGCCG<br>GAACCGGGTGCAGGTCCGACCACCGATCTG<br>CTGCGTGATAGCCGTAGTCTGCGTGGTATT<br>TTTAGCAGCTTTGCAACCGGTGTTACCGTT<br>GTGACCGTTGGTGGTGATAGTCCGCATGCA<br>ATGACCGCAAATAGCTTTACCAGCGTTAGC<br>CTGGATCCGCCTCTGATTCTGGTTTGTGTT<br>GAATGTGATGCAGCAATGCATGGTAGCCTG<br>CTGGAAGTTGGTAGCTTTGGTGTTAGCGTT<br>CTGGCAGCCGATCAGCAGCATGTTGCACTG<br>CTGTATGCAAATCGTTGGCGTCCGCGTGAT<br>CCGACCCAGTTTGATCGTCCGGGTTGGGCA<br>CGTGGTGCACGTACAGGTGCACCGCTGGCT<br>CGTGGTGCCCTGGCATGGTTTGAATGTGCA<br>CTGTGGCGTGCCTATGATGCCGGTGATCAT<br>AGCATTTTTGTTGGTCGTCTGCTGACCGCA<br>GAACGTCATGATCGTCGTGATGCACTGGTT<br>TATCATAGCGGTCAGTTTCGTGGTCTGCCG<br>GATCGTGCACCGGTTGAATAACTCGAG

SUPPLEMENTARY MATERIAL REFERENCES

1. Dong C, Flecks S, Unversucht S, Haupt C, van Pee KH, Naismith JH. Tryptophan 7-halogenase (PrnA) structure suggests a mechanism for regioselective chlorination. *Science.* **2005**;309(5744):2216-9. doi: 10.1126/science.1116510. PubMed PMID: 16195462; PMCID: PMC3315827.
2. van den Heuvel RH, Westphal AH, Heck AJ, Walsh MA, Rovida S, van Berkel WJ, Mattevi A. Structural studies on flavin reductase PheA2 reveal binding of NAD in an unusual folded conformation and support novel mechanism of action. *J Biol Chem.* **2004**;279(13):12860-7. Epub 20031231. doi: 10.1074/jbc.M313765200. PubMed PMID: 14703520.
3. Morrison E, Kantz A, Gassner GT, Sazinsky MH. Structure and mechanism of styrene monooxygenase reductase: new insight into the FAD-transfer reaction. *Biochemistry.* **2013**;52(35):6063-75. Epub 20130820. doi: 10.1021/bi400763h. PubMed PMID: 23909369; PMCID: PMC3830598.
4. Kim SH, Hisano T, Iwasaki W, Ebihara A, Miki K. Crystal structure of the flavin reductase component (HpaC) of 4-hydroxyphenylacetate 3-monooxygenase from *Thermus thermophilus* HB8: Structural basis for the flavin affinity. *Proteins.* **2008**;70(3):718-30. Epub 2007/08/31. doi: 10.1002/prot.21534. PubMed PMID: 17729270.
5. Lawrence AD, Deery E, McLean KJ, Munro AW, Pickersgill RW, Rigby SE, Warren MJ. Identification, characterization, and structure/function analysis of a corrin reductase involved in adenosylcobalamin biosynthesis. *J Biol Chem.* **2008**;283(16):10813-21. Epub 20080208. doi: 10.1074/jbc.M710431200. PubMed PMID: 18263579.
6. Webb BN, Ballinger JW, Kim E, Belchik SM, Lam KS, Youn B, Nissen MS, Xun L, Kang C. Characterization of chlorophenol 4-monooxygenase (TftD) and NADH:FAD oxidoreductase (TftC) of Burkholderia cepacia AC1100. *J Biol Chem.* **2010**;285(3):2014-27. Epub 20091113. doi: 10.1074/jbc.M109.056135. PubMed PMID: 19915006; PMCID: PMC2804359.
7. Ashaduzzaman M, Lingkon K, De Silva AJ, Bellizzi JJ, III. Crystallographic and Thermodynamic Evidence of Negative Coupling in the Flavin-Dependent Tryptophan Halogenases AbeH and BorH. *ACS Omega.* **2025**;10(6):5849-65. Epub 20250108. doi: 10.1021/acsomega.4c09590. PubMed PMID: 39989782; PMCID: PMC11840605.
